# A Qualitative Exploration of the Acceptability of a Supported Self‐Management Intervention for People With Type 2 Diabetes and Severe Mental Illness

**DOI:** 10.1155/jdr/2703200

**Published:** 2026-06-19

**Authors:** C. Carswell, J. V. E. Brown, N. Siddiqi, P. A. Coventry

**Affiliations:** ^1^ Department of Health Sciences, University of York, York, UK, york.ac.uk; ^2^ School of Medicine and Population Health, University of Sheffield, Sheffield, UK, sheffield.ac.uk; ^3^ Hull York Medical School, York, UK, hyms.ac.uk; ^4^ Bradford District Care NHS Foundation Trust, Bradford, UK; ^5^ School of Nursing and Public Health, Manchester Metropolitan University, Manchester, UK, mmu.ac.uk

## Abstract

**Background:**

People with severe mental illness (SMI) are twice as likely to develop Type 2 diabetes (T2D) compared with people without SMI and have poorer outcomes from T2D. Research evaluating self‐management interventions for T2D consistently excludes individuals with SMI, therefore existing offers fail to address the specific needs of this population. The DIAMONDS programme (Diabetes and Mental Illness, Improving Outcomes and Self‐management) developed a bespoke supported self‐management programme for people with coexisting SMI and T2D.

**Methods:**

As part of a mixed‐methods feasibility study we collected and analysed qualitative data to explore the acceptability of the DIAMONDS intervention. Semi‐structured interviews were conducted with 12 service user participants with SMI and T2D, and 10 DIAMONDS Coaches who delivered the intervention. Interviews were informed by the theoretical framework of acceptability and were thematically analysed.

**Results:**

Four overarching themes were identified, these included: Recognising and addressing a need—highlighting the importance of the intervention in the context of current gaps in care; ‘It’s all about person‐centred, essentially, isn’t it?’—emphasising the role of flexible, individualised delivery in acceptability; Utility of different intervention components—describing how different components were used; Tangible change and beneficial effects— improvements in behaviour and health, including smoking cessation.

**Discussion:**

The DIAMONDS intervention was acceptable for people with SMI and T2D and for DIAMONDS Coaches who were delivering it. A person‐centred approach enabled engagement and self‐reported behaviour change, but participants needed differing levels of support for behaviour change. These findings informed refinements to the intervention, which will be evaluated in a definitive trial.

## 1. Introduction

Type 2 diabetes (T2D) is two to three times more common amongst people who have severe mental illness (SMI), conditions such as schizophrenia and bipolar disorder, compared with those who do not have SMI [1]. People with SMI are also more likely to have poor outcomes, leading to higher rates of complications, morbidity and mortality [2]. This disparity contributes to an inequality known as the ‘mortality gap’, which describes that people who have SMI die, on average, 15–20 years earlier than those without SMI [3–5].

There are multiple contributing factors to the mortality gap. Conditions included under the SMI umbrella are associated with a profound impact on day‐to‐day function and require lifelong management and treatment, including medication regimens, social support, and psychological interventions [6]. Antipsychotic medications used to treat SMI have a constellation of side effects that can cause long‐term physical consequences, such as weight gain and metabolic syndrome [7]. Some symptoms of SMI can make it difficult for people to appreciate the risks associated with long‐term conditions and understand the importance of looking after their physical health [6, 8, 9]. Additionally, there are other inequalities that people with SMI experience, including social deprivation as people with SMI are more likely to be unhoused, unemployed and living below the poverty line than people without SMI [10], stigma and victimisation [11, 12] and social isolation, as people with SMI are less likely to have access to a social support system, or family support, compared to people without SMI [13, 14]. These inequalities are compounded by difficulties accessing healthcare services, where people with SMI struggle to make and attend appointments [6, 8, 15] and experience diagnostic overshadowing [16, 17].

This combination of factors contributes to the difficulties people with SMI have with self‐management of their physical health [6, 9, 18]. The term self‐management refers to the different behaviours people engage in to manage their health, such as being physically active or eating healthily [19]. Self‐management is essential in the care of T2D. There is evidence demonstrating that self‐management interventions are effective at improving diabetes outcomes for the general population [20]. However, these studies frequently exclude those with SMI [21, 22], and existing self‐management interventions have not been developed to address the complex issues experienced by people who have SMI [6, 8]. This is of particular concern as people with SMI experience significantly higher levels of diabetes related distress, depression, and lower quality of life compared to people with T2D who do not have SMI [23]. SMI is included within the National Diabetes Audit to identify and monitor inequality in provision of care and diabetes outcome in this population [24].

DIAMONDS [25] (Diabetes and Mental Illness, Improving Outcomes and Self‐management) is a programme of research that aims to address this stark inequality by developing and evaluating a bespoke supported self‐management intervention for people with SMI and T2D. This article presents our qualitative exploration of the acceptability of the DIAMONDS intervention within a mixed‐methods feasibility study [26].

## 2. Methods

### 2.1. The DIAMONDS Intervention

**Figure 1:**
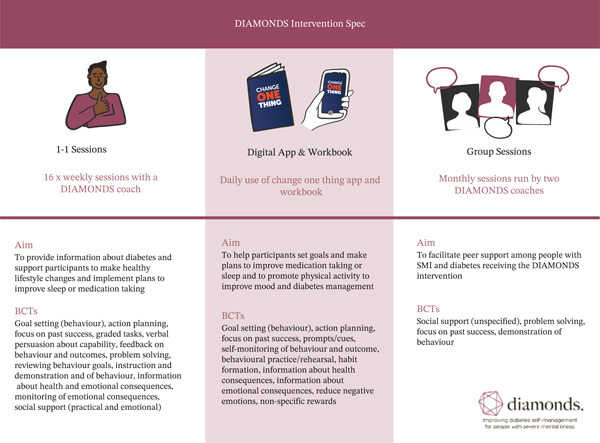
Intervention specification, including behaviour change techniques delivered through each intervention component.

The DIAMONDS intervention was codesigned with people with SMI and T2D, their carers, and healthcare professionals who support their care [27]. The intervention aims to support people with SMI and T2D to self‐manage their health by supporting them to engage in healthy behaviours and sign‐posting them to relevant support services. The intervention is delivered by DIAMONDS Coaches, who are specially trained, experienced healthcare professionals. An overview of the intervention is shown in Figure 1.

### 2.2. Design

As part of an exploratory mixed methods process evaluation within the feasibility study, we carried out semi‐structured qualitative interviews with participants and DIAMONDS Coaches to explore the acceptability of the intervention [28, 29]. Ethical approval was obtained from the Research Ethics Committee Leeds West (reference: 21/YH/0059). Intervention delivery and data collection took place between November 2021 and June 2022.

The methods and main results of the feasibility study are reported separately [26].

### 2.3. Feasibility Study Recruitment

Service users with SMI and T2D were identifying through database and caseload screening in mental health trusts, liaising with consultants, clinical teams, pharmacies and supported housing managers and through self‐referral directly from service users. Service users were eligible if they were over the age of 18, had a diagnosis of SMI (defined as a mental health condition that can present with psychosis, such as schizophrenia, schizoaffective disorder and bipolar disorder), and had a diagnosis of T2D. Service users were excluded if they had other forms of diabetes (such as Type 1 diabetes or gestational diabetes), lacked the capacity of provide informed consent, or were currently hospitalised and receiving inpatient care [26].

We recruited mid‐level healthcare workers (NHS Agenda for Change Band 4) with either experience of working with people with SMI or experience providing diabetes education to act as DIAMONDS Coaches and deliver the intervention [26].

### 2.4. Interview Participants

#### 2.4.1. Service Users

Thirty adults with SMI and T2D took part in the DIAMONDS feasibility study. Twenty‐three participants received at least one session of the intervention. The initial consent form for the study recorded participants′ willingness to be contacted for an interview. Twelve participants were interviewed as part of the process evaluation: 11 experienced at least one session of the intervention, and 1 participant had withdrawn without receiving an intervention session but consented to take part in an interview (Table 1).

**Table 1:** Overview of service user participant characteristics.

Demographic characteristics of service users	
Mean (SD)	
Age (years)	50.1 (7.6)
Gender, *n* (%)	
Male	7 (58.3%)
Female	5 (41.7%)
Ethnicity, *n* (%)	
Asian Pakistani	1 (8.3%)
British Somali	1 (8.3%)
White British	10 (83.3%)

#### 2.4.2. DIAMONDS Coaches

The research team contacted DIAMONDS Coaches once they had completed the delivery of the intervention to one participant. Coaches were sent a participant information sheet and consent form through post or email. They were then contacted five working days later to be invited to take part in an interview.

Ten DIAMONDS Coaches were recruited from the pool of 12 coaches who delivered the intervention in the feasibility study (Table 2).

**Table 2:** Overview of DIAMONDS Coach characteristics.

Coach number	Current post at time of interview
800100	Healthy living advisor in forensic mental health (Mental health background)
800101	Diabetes education and research facilitator (Diabetes background)
800102	Clinical research assistant (Mental health background)
800103	Diabetes education and research facilitator (Diabetes background)
800104	Staff nurse on a female acute psychiatric ward (Mental health background)
800105	Trainee advanced clinical practitioner in home treatment team (Mental health background)
800106	Perinatal community nursery nurse (Mental health background)
800107	Healthcare support worker (Mental health background)
800108	Community Psychiatric Nurse (Mental health background)
800109	Research assistant (Mental health background)
10 interviews	

#### 2.4.3. Carers

We had also intended to carry out interviews with informal carers of service‐user participants. However, we were unable to recruit any caregivers. This reflects the high level of social isolation that people with SMI experience and the lack of social support [cite]. Most participants could not identify anyone they considered an informal caregiver, relying mostly on formal carers, such as support workers. Therefore, in the definitive trial, we will aim to recruit both informal carers (where possible) and formal carers who provide regular support to those receiving the DIAMONDS intervention.

### 2.5. Data Collection

Semi‐structured interviews were carried out by CC and JB to explore the acceptability and feasibility of the intervention and study delivery. Topic guides (S1) were structured using the theoretical framework of acceptability (TFA) [30], which allows for a focused and theory‐based investigation of acceptability and related constructs.

The interviews took place over the phone or video call. One participant required the support of a residential worker to complete the interview, due to additional communication needs. This residential support worker previously supported the delivery of the intervention. The length of interviews ranged from 15 to 79 min.

### 2.6. Data Analysis

The semi‐structured interviews were recorded, transcribed verbatim and thematically analysed. The analysis process was supported using NVivo Version 12 [31]. The analysis was guided by the TFA, which consists of seven core components: affective attitude, burden, opportunity costs, intervention coherence, ethicality, perceived effectiveness and self‐efficacy [30]. Descriptive codes from the data were generated for the service user and DIAMONDS Coach interviews separately by CC. Following initial descriptive coding, the two data sets were merged into an overall coding set. These codes were reviewed by JB and PC and organised into the relevant TFA domains. Finally, these domains were revised into a final set of overarching themes. These themes were agreed upon by CC, PC, and JB.

### 2.7. Reflexivity

CC is a research fellow and registered mental health nurse who delivered the intervention during the feasibility study. CC did not interview the participant they had worked with during the study. JB is a research fellow with a background in health psychology. PC is a professor with experience in applied health research, especially in multiple long‐term conditions. CC, PC, and JB are mixed‐methods researchers who have experience and training in qualitative research methods. CC, JB, and PC were all involved in the development of the DIAMONDS intervention.

## 3. Results

Four themes were identified following analysis of the data (Table 3).

**Table 3:** Description of overarching themes.

Theme	Description of the theme
Recognising and addressing a need	This theme captures how the DIAMONDS Intervention addressed a significant unmet need in clinical practise and the daily lives of service users, motivating people to take part.
*It′s all about person-centred essentially, isn′t it?*	This theme highlights how the flexible person‐centred delivery of the intervention was crucial to acceptability.
The utility of different intervention components	This theme encapsulates the different opinions participants had about the components of the DIAMONDS intervention, including the DIAMONDS Workbook, Change One Thing app, and the DIAMONDS Coach.
Tangible change and beneficial effects	This theme describes the different changes and benefits participants experienced as a result of receiving the DIAMONDS intervention.

### 3.1. Recognising and Addressing a Need

A key aspect of the DIAMONDS intervention that contributed to its acceptability was that it addressed an unmet need. Participants described the importance of the DIAMONDS intervention in the context of SMI and T2D, and how the intervention was designed to address an issue that was pervasive and challenging. Coaches reflected on how they frequently encountered T2D in their clinical mental health role.

‘It’s definitely something that does need research on because it remains a problem, and that’s evident in the acute wards as well. That’s coming from somebody that’s worked in acute wards with patients that do have mental health problems that are long‐standing throughout their life, and diabetes is something they definitely struggle with, especially when they do become more unwell.—DIAMONDS Coach_800104

Participants recounted how T2D was a significant issue in their lives, and how it was difficult for them to engage in self‐management behaviours, such as eating healthy or being physically active, while also coping with the symptoms of SMI.

The illness affects my day‐to‐day living. Sometimes it keeps you pinned down and you can’t get out of your house, or do things like normal people do, like go to work or drive a car… or even down to eating the wrong foods because you want to eat sugar through or keep you lifted high. You put weight on and gain weight; the illness affects me every single minute of every single day. There’s no kind of peace, there’s no peace of mind, I’m restless …the fear, it’s being like trapped in vice with someone after you, and the fear grips you.—Service user_400103

Although participants had historically struggled to change their behaviours, they recognised that their physical health difficulties required behaviour change.

I think it’s important to change, because it’s just…we can’t expect to…those that are diabetic, can’t expect to actually live so much of a structured life with diabetes and not change the way, or try and find a way and change things to actually be a little bit better.—Service user_100104

Understanding the need to change their behaviour influenced participants’ motivation to engage in the intervention. Although participants described different goals they wanted to achieve, like improving sleep, managing medication side effects or losing weight, this motivation was consistently related to health improvements.

I’m a little bit overweight, you know, so it was helping me quite a lot because I was concerned about my diet for a while, so doing that, it’s made me look at what I’m eating and things.—Service user_600102

Coaches found that the participants felt grateful for the support they received during the intervention, which was framed as a response they did not typically receive from people they worked with. This suggests that the DIAMONDS coach role was providing necessary and welcome support that had previously been missing in participants’ care.

The feedback I got from both of them was really positive. It was nice to have someone say thank you, actually.—DIAMONDS coach_800100

**It’s all about person-centred, essentially, isn’t it?**

The person‐centred, flexible delivery of the DIAMONDS intervention kept participants engaged after enrolling in the study. Participants were encouraged to identify and develop goals and action plans alongside the DIAMONDS Coach, based on their priorities and ability to make gradual changes. Coaches described how this was a key to successful engagement, and that a standardised approach would have been inappropriate.

This person just needed someone to listen. And I think my mindset has changed a little bit on it from the beginning, where I’ve got a checklist here, I’ve got to tick it all off, it just doesn’t work like that.—DIAMONDS Coach_800103

DIAMONDS Coaches used this person‐centred approach to identify priorities and tailor behaviour change. This included identifying barriers that hindered goal achievement and devising creative strategies to overcome these.

We worked out some exercises that I could do in the house, because I don’t go out on my own, so it wasn’t like I could just go for a walk. We worked out a way that I could exercise while I was in the house, putting music on and dancing to it, doing squats while you’re washing up. We tried looking into different exercises, but I was always, oh, I’ll do them later, I’ll do them later, and I wasn’t doing them. So, we’ve worked out a way to incorporate them into whatever it was I was doing with the housework.—Service user_500104

This flexibility also extended to the mode of delivery of the intervention, meaning participants could engage in face‐to‐face or remote sessions. Some participants preferred face‐to‐face sessions, as they helped them develop rapport with their coach. However other participants preferred remote sessions to reduce the likelihood of spending time travelling.

We used Zoom… This made it easier for both of us really, it saved my Coach having to come out and he’s based in [city] and traffic can be an absolute nightmare, so instead of him having to risk getting stuck in traffic and all…and thereby waste time as well that he could be spending usefully in the office, we’re going to Zoom calls because I’m quite happy with Zoom.—Service user_200102

This need for a person‐centred, flexible approach was further emphasised by the diversity of participants. The coaches described how participants had different levels of health literacy; therefore, the coaches tailored their approach. The coaches highlighted how this person‐centred approach felt different to their typical practise and aligned more closely with their values.

Which for me, having worked for the NHS for almost 30 years, that’s a revelation for people to actually be treated as a person with their issues instead of as an issue and the person’s just an afterthought.—DIAMONDS Coach_800101

### 3.2. The Utility of Different Intervention Components

The DIAMONDS intervention consisted of several components, including the workbook, the Change One Thing app, and the coach. The coaches used the workbook to guide the sessions, identify goals, develop action plans and deliver education on T2D. The coaches described the workbook as easy to follow and an effective way of organising the sessions to keep track of progress. Participants also described using the workbook independently between sessions, with some continuing to use it once the intervention ended.

She said, are you going to keep on doing it when me and her finish and that. And there’s some bits that I carry on doing… there’s sections in it where it’s, you know, this week I’m going to focus on this, and to help me I’m going to do this.—Service user_500104

The intervention incorporated repetitive elements to facilitate the establishment of new habits and the understanding of new information. However, this repetition was not always viewed positively. Several coaches commented that some of the repetition was unnecessary and disrupted the flow of the sessions. However, other aspects of repetition were recognised as important to ensure participants remembered core concepts,

It’s basically doing the same thing twice and you’re sort of going, oh haven’t we just… talked about that? I think there’s …just a slight little tweak might be needed in that aspect of it… that repetitive sort of nature also helps reinforce things in people’s minds. So, it sort of reminds them. It’s about keeping these ideas fresh in their mind. They might not be doing them… but it plants a seed.—DIAMONDS Coach_800100

Participants and coaches were reluctant to use the Change One Thing App. Coaches and participants both described how they preferred the workbook, either because it was easier to use or because they were not comfortable using digital applications on their phones. The coaches described issues with digital literacy and remote delivery as significant barriers to engaging with the app. Participants receiving their one‐to‐one sessions over the phone could not simultaneously download, set up or use the app. As a result, the DIAMONDS workbook was the preferred way to engage,

*My chap did download his app, but there’s also that area—where a lot of people use their phone to talk to you, so they are unable to use the app. I think I could have got him to use the app if he wasn’t having these sessions on the phone. So, he ruled it out straightaway; he would not think about it.—DIAMONDS Coach_800101.*

The DIAMONDS Coach role was a key component of the intervention, ensuring it was both acceptable and enjoyable to participants. The rapport with the coach was crucial to identifying goals and developing action plans, as participants needed to feel comfortable speaking openly about their lives and priorities.

*I’d sit and talk to her about stuff that I had not talked to anybody about for years. She was really easy to get on with. And she’s always asked me before the session ended, is there anything you want to focus on next time.—Service user_500104.*

The role of the DIAMONDS Coach also extended to assuaging anxiety and building the participant’s confidence, to support them to make changes and achieve their goals by providing encouragement and reassurance. This was particularly important for people who struggled with the symptoms of SMI.

I can remember being in the café, with real anxiety, I got real bad anxiety … [the coach] wasn’t picking up her phone or anything, and I was sending her all these messages. And then when she got the message, she phoned me and said, I’ll meet you in the café, and stay in the railway station, ‘cause I was stressed out and had anxiety…. she just put me at peace straightaway.—Service user_400103

### 3.3. Tangible Change and Beneficial Effects

Participants experienced changes to their behaviour, health and knowledge through the DIAMONDS intervention. Some participants reported making sustainable behavioural changes.

Yeah, eating, exercise, reduce smoke, which I stopped now. I don’t smoke anymore.—Service user_400102

Participants also described some notable benefits from the changes made during the intervention. Supporting participants to prioritise their own goals meant they were able to address issues that were most prominent in their lives and therefore had the most benefit.

I wasn’t sleeping on a night, I couldn’t sleep, I’d be up till really late, and I wouldn’t be able to calm down, you know, where I was really struggling to calm myself down and that, which as soon as I get worked up my hallucinations would get worse. I was shattered because I hadn’t had a proper night’s sleep. And in the space that I work in with the DIAMONDS Coach we got my sleeping more or less back to normal.—Service user_500104

Participants also gained new knowledge through the DIAMONDS intervention, describing how the intervention ‘gives people insight into mental illness and diabetes’. Although participants’ priorities generally focused on mental health, this new understanding helped them link improving their physical health to improvements in their mental health. As a result, they developed goals and action plans around physical activity and healthy eating, acknowledging that this would have beneficial effects on their mood,

When I do exercise… I’m feeling some, how can I say, I feel happy… Yeah, I feel happy.—Service user_400102

The DIAMONDS Coaches identified components of the intervention that contributed to the benefits participants experienced. The coaches described how the relationship they had with participants, with regular support and patience, facilitated behaviour change. There was an acknowledgement that change could only occur when the participant was ready; therefore, consistent input was essential.

Just having space to talk and share and start to… It depends on where someone is, doesn’t it, on that cycle of change and sometimes you’re working with them when they’re not ready at times. So, it really depends. And I’ve realised week‐by‐week that does change as well.—DIAMONDS Coach_800103

The coaches felt some participants may benefit more from the intervention than others. The coaches explained that several participants who took part were already effectively managing their T2D before the intervention, and it was unlikely they experienced many benefits. Instead, they felt that those who were struggling stood to benefit the most,

Probably with people who find it hard to manage their diabetes in general and need direction and aren’t possibly as independent as the person that I had. So maybe someone who has more health‐related problems and has quite intense symptoms from diabetes, and maybe someone… who struggles managing bipolar.—DIAMONDS Coach_800102

## 4. Discussion

The DIAMONDS feasibility study is aimed at examining the feasibility and acceptability of a bespoke supported self‐management intervention for people with coexisting SMI and T2D [29]. Both service user and coach participants expressed that the intervention was acceptable and identified key components that made it feasible to deliver. For service users these components included the person‐centred approach and the provision of a supportive coach which allowed them to successfully engage with the intervention and empowered them to change their behaviour. This is distinct from self‐management interventions aimed at the general population, where education is typically delivered in standardised group sessions [32]. The findings from these qualitative interviews identified changes that needed to be made to the intervention before evaluation in a definitive trial, such as reducing repetition of content in the workbook. The findings also confirmed that participants were reluctant to use the Change One Thing App due to their lack of familiarity with navigating digital phone applications, highlighting the issue of digital exclusion in this population [33]. Although the app was retained in the definitive trial [34, 35], the coach training was altered to better support participants to engage with the app. The app was retained to prevent compounding the issue of digital exclusion by not offering the option of a digital method of engaging in the intervention, particularly as the mean age of participants in the feasibility study was 52.3 years [cite], and may not reflect the needs of younger service users. Participants described key behaviour changes, including improved sleep, increases in physical activity and smoking cessation, all of which can contribute positively to diabetes outcomes [36–38].

The main strength of this study was the involvement of service users, carers and healthcare professionals in the development of the intervention [39], this reflects the approach of collaborative care in clinical practise, where primary care services, mental health services and specialist physical health services work collaboratively to provide support for people with coexisting mental health and physical health conditions. The interviews also included a range of DIAMONDS Coaches, with differing backgrounds and experience of mental health and diabetes care, and participants who contributed to the feasibility study. The participants who agreed to be interviewed were mainly White British, and all were over the age of 40, limiting the generalisability of findings [40]. We confirmed the diagnosis of SMI during screening for the feasibility study; however, we did not capture individual‐level data on diagnosis and we were also unable to individual‐level data on diabetes history, with only 20% of participants in the feasibility study able to provide this information through self‐report, therefore, were unable to examine how different types of SMI or history diabetes might influence the acceptability and feasibility of the intervention.

This study has demonstrated that involving service users, carers and healthcare professionals in the development of the intervention ensured that it addressed the needs of this population. Although we identified some minor changes to improve the user experience of the intervention, overall, the intervention was highly acceptable. The findings reflect how SMI influences the level of support needed and how people engage with self‐management, highlighting the importance of considering coexisting SMI when providing diabetes care within this population.

## Author Contributions

C.C. and J.V.E.B. contributed to data curation, formal analysis, investigation, project administration, resources, and writing – reviewing and editing. C.C., J.V.E.B. and P.A.C. contributed to methodology. C.C. contributed to writing – original draft. N.S. and P.A.C. contributed to conceptualisation, funding acquisition, supervision, validation, and writing – review and editing. C.C. had full access to all of the data in this study and takes complete responsibility for the integrity of the data and the accuracy of the data analysis.

## Funding

This study is part of the DIAMONDS Programme which is funded by the National Institute of Health and Care Research Programme Grant for Applied Research, NIHR PGfAR RP‐PG‐1016‐20003.

## Disclosure

All authors have read and approved the final version of the manuscript. The views expressed are those of the authors and not necessarily those of the NIHR or the Department of Health and Social Care.

## Conflicts of Interest

The authors declare no conflicts of interest.

## Supporting information


**Supporting Information** Additional supporting information can be found online in the Supporting Information section. S1 SRQR Reporting checklist.

## Data Availability

The data that support the findings of this study are available on request from the corresponding author. The data are not publicly available due to privacy or ethical restrictions.
