# Supplementary material for: A Qualitative Exploration of the Acceptability of a Supported Self‐Management Intervention for People With Type 2 Diabetes and Severe Mental Illness
Source: J Diabetes Res. 2026 Jun 19;2026:2703200. doi: 10.1155/jdr/2703200 (PMC13282270; doi:10.1155/jdr/2703200)
Supplement: Supplementary file 1 — Supporting Information Additional supporting information can be found online in the Supporting Information section. S1 SRQR Reporting checklist. [file JDR-2026-2703200-s001.zip › DIAMONDS TOPIC GUIDE NON COMPLETERS v1.0.docx]

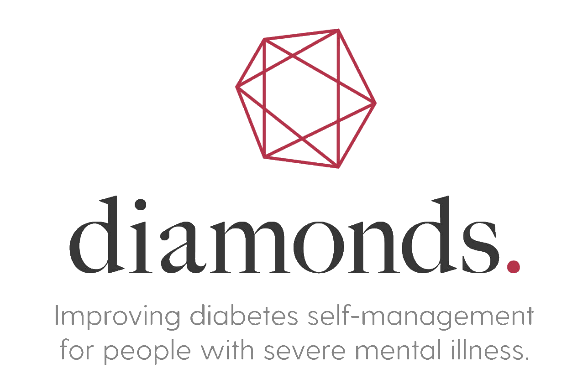


**DIAMONDS: Diabetes and Mental Illness Improving Outcomes and Services**

**TOPIC GUIDE: Participants (‘Non-Completers’)**

**COVID 19 CONTEXT, SMI AND DIABETES IMPACT AND SUPPORT**

- If I can start by asking generally if the situation with COVID has had an impact on your life? (pull out behaviour (do) as well as psychological (felt))
  - **Prompt** Have you needed to make any adaptations to try and carry on as you have previously?
  - **Prompt** Did anything make the situation easier or more difficult?
  - **Prompt** Generally, have there been any coping strategies, that it’s been possible to use ?
  - **Prompt:** Have you been shielding/social distancing?
- Has the situation with COVID had an impact on your health and wellbeing?
  - **Prompt:** Tell me how your mental and physical health were before COVID restrictions. How did you previously manage your SMI and diabetes?
  - **Prompt:** Did the restrictions have any impact on your SMI (e.g. management/treatment) (again - pull out behaviour as well as psychological)
  - **Prompt:** Did the restrictions have any impact on your Diabetes (e.g. management/treatment) (again - pull out behaviour as well as psychological)
  - **Prompt:** Did the restrictions have any impact on your ability to attend appointments/ access healthcare services?
  - **Prompt:** has there been any sources of support that have been more or less helpful generally?

(Funnel it towards **how restrictions might have made it more difficult to look after their mental and physical health) -** have a general conversation about **the perceived and real impact of the pandemic,** with an eye on steering it to management of SMI and diabetes

**TFA DOMAINS, STUDY MATERIALS & PROCESSES**

- What were your thoughts when you were first invited to participate in the DIAMONDS study?
  - **Prompt:** how were you first contacted/was the written information clear? - what about the follow-up phone call/was this okay – other ways to make contact?
- What were you expecting from participating in the DIAMONDS study?
- Why did you decide to participate?
- Generally, what are your thoughts on what the DIAMONDS study is trying to find out?
- So, thinking about the DIAMONDS support programme, what did you do with your DIAMONDS Coach? (explore what happened in the contacts and sessions with the Coach; what sort of things they did between sessions, explore any issues with telephone/possibly online delivery, (what support, advice, and suggestions were offered).
- **You didn’t continue working with the DIAMONDS Support programme – can you tell me why that was?**
- What do you think of the DIAMONDS workbook?
  - Was this understandable?
  - Was anything more or less useful about the workbook?
  - Could anything be changed?
  - What about the action planning used in the booklet – what did you think of this – did they seem helpful to you?
  - In terms of the practical use of the workbook, how did you find it to engage with, and read (e.g. colour scheme)?
  - Do you think you will use the workbook at all in the future?
- What do you think of the DIAMONDS app?
  - Was this easy to use/navigate?
  - Was this understandable?
  - Was anything more or less useful about the app?
  - Could anything be changed?
  - What about the action planning used in the app – what did you think of this – did this seem helpful to you?
  - In terms of the practical use of the app, how did you find it to engage with (e.g. colour scheme, layout, push notifications)?
  - Do you think you will use the app at all in the future?
- How much effort was needed to take part in the DIAMONDS support programme?
- **Prompt:** this is the overall effort required for meetings with your Coach and other things you did in-between sessions (This could be time/expense/mental effort)
- **Prompt:** What was more difficult /easy? – in what way /how?
- Did you have to give anything up, or not do other things so that you could participate in the DIAMONDS study? ((Could be things you might have enjoyed, time, financial cost - benefits, profits or values)
- Generally, how confident did you feel taking part in the DIAMONDS support programme?
- Were there any aspects of the DIAMONDS support programme that you felt more/less confident with.
  - **Prompt:** did this change? how/when?
- Thinking about the sessions you had with the Coach, how often did you meet with the Coach (e.g. 1 or 2x/week)?
  - **Prompt:** How was this, would you have liked to meet more or less often, or maybe it seemed about right?
- Are there any changes you have made as a result of taking part in the DIAMONDS support programme?
  - **Prompt:** Have you been able to maintain these changes? (explore reasoning)
  - **Prompt:** Have these changes helped you manage your diabetes/ SMI?

**REFLECTIONS ON THE STUDY OVERALL AND THE TFA DOMAINS**

- Generally, how do you feel about taking part in the DIAMONDS study?
- Would you recommend the DIAMONDS support programme to others?  why? who?
- Overall, do you think the DIAMONDS support programme will be able to achieve its aims, and be of help?
  - **Prompt:** if so why/if not why not?
- Was there anything more of less helpful about receiving the DIAMONDS support programme?
- Is there anything that you valued most about the DIAMONDS support programme? / Least valued - why?
- Do you think anything could be done differently, or improved?
  - (**Prompt:** this might be how the DIAMONDS support programme is delivered, or the content of the programme)
- Is there anything else that perhaps I have not asked you about, but that you would like to say?
