# Supplementary material for: A Qualitative Exploration of the Acceptability of a Supported Self‐Management Intervention for People With Type 2 Diabetes and Severe Mental Illness
Source: J Diabetes Res. 2026 Jun 19;2026:2703200. doi: 10.1155/jdr/2703200 (PMC13282270; doi:10.1155/jdr/2703200)
Supplement: Supplementary file 1 — Supporting Information Additional supporting information can be found online in the Supporting Information section. S1 SRQR Reporting checklist. [file JDR-2026-2703200-s001.zip › DIAMONDS TOPIC GUIDE COACH v1.0.docx]

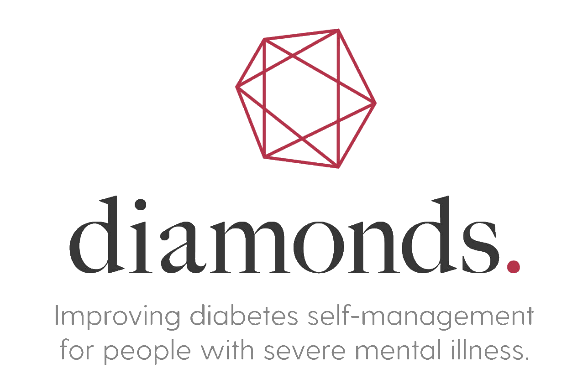


**DIAMONDS: Diabetes and Mental Illness Improving Outcomes and Services**

**TOPIC GUIDE: DIAMONDS Coaches**

Please could you outline your usual role?

**Prompt:** How long have you been in that role? – In your working area in general?

**COVID 19 CONTEXT, SMI AND DIABETES**

- Generally how has it been working with people with SMI and diabetes in the context of the restrictions due to COVID 19 (generally first if they currently work with people with SMI - and in the DIAMONDS study)
- What sort of impacts do you think the restrictions have had on people with SMI in general?
- What sort of impacts do you think the restrictions have had on people living with both SMI and diabetes?
- How do you think participants have been coping (strategies/differences)
- What sort of adaptions have they needed to make to their lives in order to cope with COVID restrictions?
- Do you think the restrictions have affected the ways in which people with SMI are coping/managing their SMI?
- Do you think the restrictions have affected the ways in which participants are coping/managing their diabetes?
- What are the main challenges in working with people with SMI and diabetes?
  Generally what are the main challenges in working with people with SMI and diabetes in the current context?

**TFA DOMAINS, TRAINING and ASSOCIATED MATERIALS & STUDY PROCESSES**

- How did you become involved in the DIAMONDS study?
  - **Prompt:** what were your thoughts when you were asked to deliver the DIAMONDS support programme?
- Why do you think someone might decide to participate in delivering the DIAMONDS study? - What about yourself?
- What were your expectations of the study?
- What are your thoughts around the main aims of the DIAMONDS study?
- How was the DIAMONDS Coach training?
- What aspects worked well?
- What worked less well or could be done differently? (explore online format)
- Are there any areas that you think would be useful to add or modify in the Coach training?
  - **Prompt:** training around SMI / Diabetes/ Behaviour change)
- How useful was the Coach manual you were given to support your delivery of the intervention?
  - How/when did you use the Coach manual?
  - How was it using the add-on appendices in the Coach manual for the DIAMONDS study?
  - Are there any parts you seemed to use more or less than others?
  - Could any changes be made?
- How was it using the DIAMONDS website for the DIAMONDS study?
  - How/when did you use the DIAMONDS website?
  - Are there any parts you seemed to use more or less than others?
  - Could any changes be made?
- How did you find communication with the research team?
- What aspects of communication worked well?
- What worked less well or could be done differently?
- Did you need to report a participant withdrawal or adverse event to the research team?
  - **If YES:** How did you find that process?

**DIAMONDS CONTEXT, INTERVENTION DELIVERY AND ACCEPTABILITY**

- How did you find working with participants with SMI?
- Any issues?
- What about diabetes?
  - **Prompt:** challenges/enablers for intervention delivery (were there any issues around either SMI or diabetes self-management/monitoring in this context)
- Please could you describe how study participants linked their ability to self-manage their SMI with their ability to self-manage diabetes?
  (**prompt:** coping with both mental and physical health conditions)
- Thinking about how the support programme was delivered, what were the advantages/disadvantages of telephone/online sessions with study participants?
- What is your general understanding about how the DIAMONDS support programme is proposed to work?
  - **Prompt –** for example for participants
- How did you find using the workbook with participants? (What was useful, or less useful – for you /participants?)
  - What are your thoughts about the action planning used in the booklet?
  - What are your thoughts about the diabetes education provided in the booklet?
  - Could the booklet be modified in anyway?
- How did you find using the ChangeOneThing app with participants? (What was useful, or less useful – for you /participants?)
  - What are your thoughts about the action planning used in the app?
  - What are your thoughts about the monitoring function of the app?
  - Could the app be modified in anyway?
- How did you find monitoring people’s self-management behaviours? (if uncomfortable, explore why)
- Did any COVID 19 restrictions impact on your ability to deliver the DIAMONDS support programme and support people to make changes?
- How did the people you worked with respond to the support programme?
- Did you have any people who did not complete the support programme? – why was that do you think?
- Do you think the support programme seemed to work better for some people rather than others?
- What sort of people do you think the support programme worked for? (or didn’t work for?)

**GENERAL REFLECTIONS ON THE STUDY AND TFA DOMAINS**

- Generally, how do you feel about delivering the DIAMONDS support programme?
- What sort of effort was needed to deliver the DIAMONDS support programme?
  - **Prompt:** could be time, expense, cognitive effort -
  - what was more difficult /easy? – in what way /how?
- How confident did you feel undertaking the delivery of the support programme?
  - **Prompt:** did this change – how /why?
- Were there any aspects of the study you felt more/less confident with.
- What were they?
- Did you have to give anything up, or not do other things so that you could participate in delivering the DIAMONDS study? (could be benefits, profits or values)
- What were the difficulties (if any) associated with working in the DIAMONDS study? (explore any concerns working in covid19 pandemic)
- Overall, do you think the DIAMONDS support programme will achieve its purpose?
  Prompt: if so why/if not why not?
- Is there anything that you valued most about the DIAMONDS study?
  - **Prompt**: least valued - why?
- Would you recommend the DIAMONDS study to others?
  - **Prompt:** Perhaps potential Coaches/ People with SMI and diabetes
- Who? Why?
- Is there anything else you would like to say, perhaps that I have not asked you about?
